# Evaluation of Th2 and Th17 Immunity-Related Factors as Indicators of Brucellosis

**DOI:** 10.3389/fcimb.2021.786994

**Published:** 2022-01-07

**Authors:** Reza Gheitasi, Fariba Keramat, Sara Khosravi, Mehrdad Hajilooi, Mathias W. Pletz, Oliwia Makarewicz

**Affiliations:** ^1^ Institute for Infectious Diseases and Infection Control, Jena University Hospital, Jena, Germany; ^2^ Department of Immunology, School of Medicine, Hamadan University of Medical Sciences, Hamadan, Iran; ^3^ Brucellosis Research Center, Hamadan University of Medical Sciences, Hamadan, Iran; ^4^ Department of Microbiology, School of Medicine, Hamadan University of Medical Sciences, Hamadan, Iran

**Keywords:** *Brucella melitensis*, adaptive immunity, cytokines, lncRNA, qPCR, arthralgias

## Abstract

**Objective:**

Brucellosis is a common bacterial zoonotic infection, and greater than half a million new cases are diagnosed annually. This study investigates the expression of Th2 and Th17 immunity-related factors (Th2-LCR lncRNA, IL-25, TRAF3IP2, and IL-17RB) in different stages of *Brucella* infections.

**Material and Methods:**

In total, 99 brucellosis patients were divided into three groups (*acute* = first infection before treatment, *relapse* = before treatment, and *treated* = after treatment for 6–8 weeks with doxycycline and rifampin). Thirty-three healthy volunteers represented the *control* group. Gene expression levels were assessed by quantitative amplification in reference to the 18S rRNA gene and statistically evaluated.

**Results:**

No significant differences in the expression of these genes were observed between the control group and patients after completion of antibiotic treatment. Compared to these two groups, only Th2-LCR lncRNA and TRAF3IP2 were significantly more highly expressed in the acute group. Th2-LCR lncRNA was also significantly elevated in the relapse group. TRAF3IP2 expression was additionally significantly increased in the acute group compared to the relapse group.

**Conclusion:**

IL-25 and IL-17RB failed to differentiate between the infected and noninfected groups. TRAF3IP2 and Th2-LCR lncRNA might be good indicators of brucellosis during the acute phase, but the expression levels varied strongly among patients. To verify the suitability of these factors as an indicator for brucellosis, acute infection or relapse should be investigated in further studies on larger cohorts with well-defined inclusion criteria.

## Introduction

With greater than half a million new cases per year (De Figueiredo et al., 2015), brucellosis is one of the most common zoonotic infectious diseases worldwide. Brucellosis is caused by Gram-negative, intracellular bacteria of the *Brucella* genus. Infections in both humans and animals typically occur by the oral or inhalation route *via* mucous membranes (Kaufmann et al., 1980; Dadar et al., 2019). Innate immune cells represent the first line of defense and try to eliminate invading pathogens. However, brucellae can overcome this defense by hiding and replicating in mononuclear cells (Rittig et al., 2001; Watarai et al., 2002), which spread the pathogen *via* the lymphatic system to various organs (Baldwin and Winter, 1994; Hasanjani Roushan et al., 2004). This condition manifests in a variety of nonspecific symptoms, varying from mildly acute to chronic (Rafiei et al., 2006), and the condition is even fatal in isolated cases (Carrington et al., 2012).

To date, known intracellular signaling cascades in brucellosis have been described in detail in several reviews elsewhere (De Figueiredo et al., 2015; López-Santiago et al., 2019; Ferrero et al., 2020). Briefly, innate antigen-presenting cells (APCs) activate naïve CD4^+^ T helper (Th) cells and CD8^+^ cytotoxic T (Tc) cells. The primed CD4^+^ Th cells further differentiate into Th1 cells or Th2 cells in response to the specific cytokine milieu provided by activated APCs. Activated macrophages and Th1 cells control *Brucella* infection mainly by IFNγ secretion, which further stimulates the cellular response (Giambartolomei et al., 2002; Brandão et al., 2012) *via* CD8^+^ cytotoxic T cells of type 1 (Tc1) against *Brucella*-infected cells (Skendros and Boura, 2013; Rahmanpour et al., 2019). During the progression of infection, humoral immunity increases upon stimulation by IL-4-producing CD4^+^ Th2 cells, which mediate the activation of B cells and subsequent antibody production (Zhu, 2015). Natural killer (NK) cells also appear to play an important role in the pathogenesis of brucellosis through the early production of IFNγ and their cytotoxicity, which has been shown to be inhibited during acute brucellosis (Salmerón et al., 1992). NK cells may also induce antibody production by B cells (Gao et al., 2011).

During intracellular infections, Th17-mediated immunity (often associated with autoimmune diseases) has also been shown to be activated, bridging and modulating both the Th1 and Th2 responses by IL-25 (also known as IL-17E) (Bai et al., 2009). IL-25 is produced by damaged epithelial cells or activated mast cells, macrophages, eosinophils, and Th2 cells and is mainly associated with allergic diseases (Besnard et al., 2011). IL-25 receptors (composed of IL-17RA and IL-17RB subunits) are present in both NK cells and CD4^+^ Th cells (Terashima et al., 2008; Stock et al., 2009). The signaling cascades initiated from the receptors of the IL-17 family are mediated by TRAF3 interacting protein 2 (TRAF3IP2, also known as Act1), a key upstream activator of various inflammatory mediators. IL-25 can suppress Th1 and Th17 immune responses by inhibiting IL-12 or inducing IL-23 production, respectively, reducing the tissue-damaging effects of inflammation (Caruso et al., 2009). IL-25 also increases the secretion of Th2-related cytokines (including IL-4), stimulating the humoral response. IL-4 is encoded in one gene cluster that includes genes for IL-5 and IL-13 and a locus control region (LCR) (Chang et al., 2006) that regulates the expression of these cytokines (Zeng, 2013). The LCR overlaps a long noncoding (lnc)RNA (hereafter referred to as Th2-LCR-lncRNA) that is predominantly expressed in primary and effector Th2 cells (Spurlock et al., 2015) and positively regulates the transcription of IL-4, IL-5, and IL-13. The Th2-LCR lncRNA profile can be altered by pathogens, thereby influencing the immune response and pathogenicity (Wen et al., 2020).

We investigated the expression of IL-25, IL-17RB, Th2-LCR lncRNA, and TRAF3IP2, which play roles in brucellosis. The aim of this study was to assess the potential of these Th2 and Th17 factors as markers for the infection status defined as acute or relapse. To the best of our knowledge, this study represents the first preliminary data on TRAF3IP2 and Th2-LCR lncRNA expression in humans suffering from brucellosis.

## Materials and Methods

### Ethical Approval and Patient Consent

This study was approved by the local ethical committee of the Hamadan University of Medical Sciences under reference number IR.UMSHA.REC.1397.760. All participating patients and volunteers were informed about the study, applied procedures and any risks due to sampling by a physician and provided their written consent. Participants were also informed that they could withdraw from the study at any time.

### Sample Collection and RNA Extraction

Blood samples were collected in 10-ml vacutainer tubes containing EDTA (BD Vacutainer, USA) from patients and volunteers upon written consent between July 2018 and April 2019. Total RNA was extracted using RNX-plus solution (CinnaGene, Iran, RN7713C) following the manufacturer’s instructions. The concentration and purity of extracted RNA were analyzed using a Nanodrop (A&E Lab, Nano200, UK), and the integrity of total RNA was controlled by gel electrophoresis in a 1% agarose gel supplemented with 2.2 M formaldehyde in MOPS-buffer.

### Reverse Transcription Reaction and qPCR

Primers (**Supplementary Material, Table S2**) were designed for the LCR-lncRNA, IL-17E, IL17-RB, TRAF3IP2, and 18S rRNA genes. The primers were provided by the Bioneer company (South Korea) in lyophilized form and resuspended in DEPC-treated water to a final concentration of 10 µM and stored at −20°C.

cDNA synthesis and quantitative PCR (qPCR) were performed using 1 μg of total RNA as previously described in triplicate (Gheitasi et al., 2020). Signals reaching the threshold within 36 cycles were considered for quantification. The △Ct values of each target were assessed in relation to 18S-rRNA. The qPCR efficiency for all primer pairs was Ø 2.01 ± 0.05 (between 1.97 and 2.0) and thus almost identical. Therefore, the relative fold change in transcripts of the targets was calculated based on the 2^−△△Ct^ in relation to the mean of the control group for all individual patients (including control group).

### Statistical Analysis

GraphPad Prism version 8 (GraphPad Software, USA) was applied for all statistical analyses. The normality of variables was assessed using the Shapiro–Wilk test. To compare the groups, two-way ANOVA with Bonferroni’s multiple comparison test was performed for the 2^−△△Ct^ values. A *P*-value less than 0.05 was considered significant. Correlations between all pairs of datasets were analyzed using Pearson’s rank test.

## Results

### Study Population and Clinical Features

Patients referred to the Infectious Diseases Centre of Sina Hospital (Hamadan, Iran) with suspected brucellosis who showed clinical manifestations, such as fever (<37°C) or chills, subjective weight loss, headache, and body pain (namely, myalgia, bone and/or joint pain), were eligible for the patient groups. Subjective manifestations were collected using a questionnaire. The diagnosis of brucellosis was confirmed by serological tests, including the Wright test (titer ≥1/80) and 2-mercaptoethanol (2-ME) test (titer ≥1/40), and approved by infectious disease specialists. In total, 99 patients (67.67% male and 32.32% female) with brucellosis were enrolled into three patient subgroups (each of 33 patients) that were defined as follows: I) Acute: patients with positive serological tests and related symptoms for the first time and before treatment. II) Treatment: patients with symptoms and clinical manifestations directly after they received routine treatment (100 mg/BD doxycycline and 600 mg/day rifampin) for four to eight weeks for the first time. III) Relapse: patients who displayed clinical symptoms between three months and two years after first routine treatment and not under treatment with a second therapy to date. Additionally, a control group of 30 individuals selected based on negative serological tests and history of other infectious diseases was included. Pregnant women and patients with other infectious diseases, cancers or autoimmune diseases were excluded.

The descriptive analysis of this study cohort (**Supplementary Material, Table S1**) was already published in a previous work, where other markers (cMAF and Linc-MAF-4) were investigated (Gheitasi et al., 2020). Briefly summarizing, within the patient subgroups, the sex and age distribution were similar with twice as many male cases and an average age of 45.96 ± 15.36 years. In the control group, gender was equally distributed, and the average age was slightly lower at 41.03 ± 9.48. According to clinical characteristics, most patients suffer from fever, chills and arthralgias (~72.1%). Other clinical manifestations exhibited among the study population included the following: weight loss (31.3%), headache (46.4%), and body pain and myalgia (approximately 47.4%).

### Increased TRAF3IP2 and Th2-LCR Expression in Infected Patients

The expression level of the 18S rRNA gene ranked between 10.04 ± 1.858 and 11.02 ± 1.515 cycles (means and SD), passed the normality test, and did not differ significantly between the groups (P = 0.491) (**Supplementary Material, Table S3** and **Figure S1A**), indicating its suitability as a reference gene in this experimental setup.

In general, the △Ct values of the chosen 4 target genes varied more strongly than the expression of 18S-rRNA (**Supplementary Material, Figure S1B**), and some targets in some groups (IL-25 and Th2-LCR lncRNA in the relapse group and IL-17RB and TRAF3IP2 in the acute group) did not pass the normality test (**Supplementary Material, Table S3**, indicated in red). In the control group, the distribution of the △Ct values passed the normality test for all target genes.

**Figure 1:**
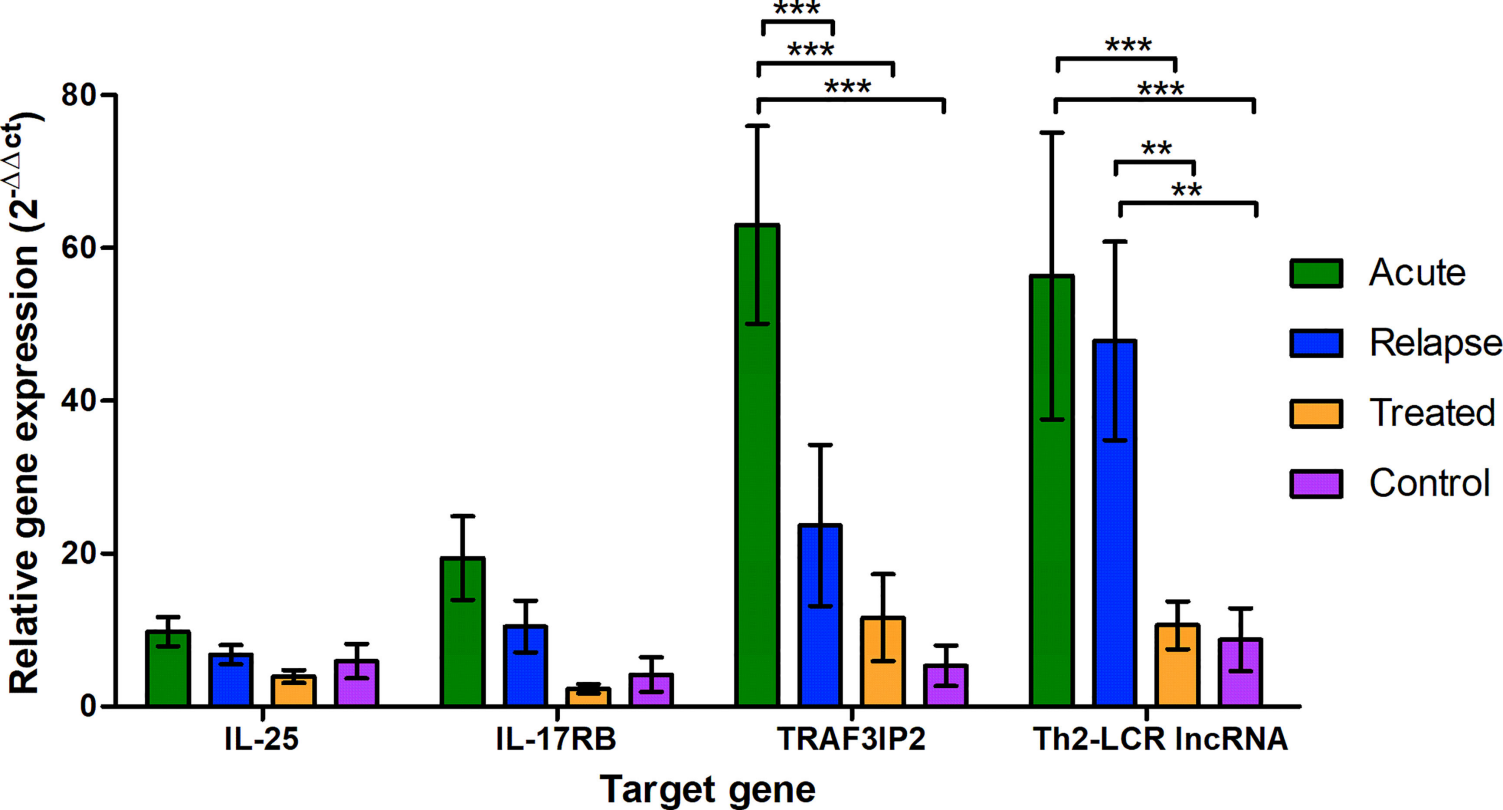
Relative expression of the selected genes (X-axis) in the different study groups (legend) shown as 2^−△△Ct^ to the reference gene (18S-rRNA) and the mean of the control group. The values are presented as the mean and the standard error of the mean (SEM). *P < 0.05, **P ≤ 0.01, and ***P < 0.001.

The control group was used to normalize and compare the changes in the expression levels of the targets between the groups. The mean △Ct value of the control groups was used for this purpose and was subtracted from all individual values, including the control group values, to visualize the variation within this group. With a percentage coefficient of variation (%CV) of >100, the standard deviation was relatively high in all groups (**Supplementary Material, Table S4**). However, the 2^−△△Ct^ of the control group was less than 20 and showed a similar pattern for all target genes (**Figure 1**). The expression patterns of IL-25 and IL-17RB did not significantly differ in the groups (**Figure 1**). Despite the high scattering of values, the expression of TRAF3IP2 and Th2-LCR lncRNA was significantly increased in the acute group, and Th2-LCR lncRNA was additionally increased in the relapse group. Compared to each other, neither expression pattern differed significantly.

## Discussion

Most studies on the immune response and modulation due to *Brucella* infection have been performed in cell-line models and animals, and the pathophysiology of this condition in humans is still not fully understood. The present study aimed to investigate specific immune factors in patients suffering from brucellosis in the acute and release phases (before antibiosis) and also in patients not receiving antibiotic treatment and to compare the expression of these factors in relation to the healthy control group.

From a different cohort from the Sina Hospital collected a year prior the present study, it was shown that Th1 and Th17 cell subsets were increased in the acute and relapse patients compared to the other two groups (Rahmanpour et al., 2019). The increased concentrations of IFN-γ in the acute and relapse groups were in agreement with the elevated Th1 cell content, but no significant increase was observed for the IL-17A that is secreted by activated Th17 cells. Simultaneously, the IL-22, as a cytokine related to defense of epithelial cells against invading pathogens was increased in acute and relapse patients providing some evidence for protective immunity against *Brucella*. Another recent study for a brucellosis cohort from China also showed increased levels of IL-17A in acute (defined as duration of disease was less than 6 months) and chronic infections (defined as not recovered for more than 6 month), but in contrast, Th17 cell counts was not significantly elevated (Zheng et al., 2019). In that study, the Th2 cell count and also IL-5, Il-4, and Il-13 were particularly elevated in chronic patients. In a previous study on the present cohort (Gheitasi et al., 2020), the expression of cMAF, a transcriptional activation e.g., of IL-10 related with the shift towards Th2 response (Cao et al., 2005), was significantly higher in the relapse group when compared to the healthy controls. These previous studies lead us to the hypothesis that there is a shift to a protective autoimmunity and humoral immune response in brucellosis. Thus, this work focused on the expression of four Th2 and Th17 immunity-related factors (Th2-LCR lncRNA, IL-25, TRAF3IP2, and IL-17RB) in different stages of brucellosis.

The current study revealed that TRAF3IP2 and Th2-LCR lncRNA expression was particularly increased in the acute phase of brucellosis. IL-25 and IL-17RB apparently failed to differentiate between the infected and noninfected groups. During relapse, TRAF3IP2 expression seemed to decrease, whereas the Th2-LCR lncRNA level remained high.

Th2-LCR lncRNA is required for the expression of IL-4, IL-5 and Il-13 and subsequently for CD4^+^ Th-cell differentiation and humoral response (Spurlock et al., 2015). The expression of Th2-LCR lncRNA has thus far not been investigated in brucellosis or other bacterial infections, and it is unclear to what extent this factor is selective for brucellosis.

TRAF3IP2 is the key activator of Th17-mediated inflammatory responses but also suppresses the humoral B cell response by negatively regulating CD40L and BAFF signaling (Qian et al., 2004). TRAF3IP2 seems to be more specific to *Brucella* infections than other Gram-negative bacteria. In the study of Degos et al., TRAF3IP2 expression was increased fourfold in human blood DCs after exposure to the *Brucella* virulence factor CβG (β-1,2 cyclic glucan) compared to the *E. coli* lipopolysaccharides (LPS) (Degos et al., 2015) CβG is a highly abundant virulence factor of *Brucella* that modulates membrane rafts of the infected cell necessary for the intracellular escape (Arellano-Reynoso et al., 2005). It is thus one of the most *Brucella*-specific toxins recognized by immune cells (Arellano-Reynoso et al., 2005; Degos et al., 2015). LPS is another important Gram-negative virulence factor. However, it is altered in *Brucella*, so it is only weakly immunostimulatory (Lapaque et al., 2005; Barquero-Calvo et al., 2007).

TRAF3IP2 might represent an interesting candidate as a potent marker that might even be useful to differentiate between patients with acute and relapsed brucellosis. However, this differentiation would require the use of an additional, well-suited reference gene from the immune cascade for normalization (e.g., based on a ratio), as the ΔCt alone fluctuated too much. In addition, comparisons with other non-brucellosis-infected groups should be performed to test for selectivity. It is possible that this factor has a more general function in specific infections (e.g., intracellular bacteria or cell damage).

The wide range of the qPCR results generally points to fundamental difficulties in quantitative analyses of patient material. Immunological parameters are difficult to differentiate cleanly, as the immune response is influenced by many individual factors that are generally difficult to define and record in the study design. The observed non-Gaussian distribution of the acute and relapse groups might result from unreliable assignment to the groups. Given that the classification ‘acute’ was based on a subjective survey of the patients, patients may have given false information against their better knowledge. For the classification ‘relapse’, the different characteristics of pathogenesis and chronification could have played roles. Therefore, in further investigations of brucellosis in patients, the inclusion criteria and group assignments should be more clearly and tightly defined. For example, it could help to include only patients whose medical history is well documented and to use standardized questionnaires that are also completed by hospital staff based on medical history and documentation. Longitudinal prospective studies, which are admittedly more difficult to conduct, would be excellent for assessing the changes in specific factors or markers in the individual course of the disease to better understand the immunological processes in brucellosis.

## Data Availability Statement

The original contributions presented in the study are included in the article/**Supplementary Material**. Further inquiries can be directed to the corresponding author.

## Ethics Statement

The studies involving human participants were reviewed and approved by the Iran National Committee for Ethics in Biomedical Research, Hamadan University of Medical Sciences (IR.UMSHA.REC.1397.760). The patients/participants provided their written informed consent to participate in this study.

## Author Contributions

Conceptualization: FK and MH. Methodology: MH. and RG. Investigation: RG. Validation: FK, MH, and RG. Formal analysis: OM and RG. Resources: MH, RG, and MP. Data curation: OM and RG. Writing—Original draft preparation: SK and RG. Writing—Review and editing: OM, RG, and MP. Visualization: OM and RG. Supervision and project administration: FK and MH. All authors contributed to the article and approved the submitted version.

## Funding

This work was supported by grants from the Vice Chancellor for Research and Technology at Hamadan University of Medical Sciences under the grant number 9710186141 and from the European Union’s Horizon 2020 research and innovation program under Marie Skłodowska-Curie grant agreement number 861323.

## Conflict of Interest

The authors declare that the research was conducted in the absence of any commercial or financial relationships that could be construed as a potential conflict of interest.

## Publisher’s Note

All claims expressed in this article are solely those of the authors and do not necessarily represent those of their affiliated organizations, or those of the publisher, the editors and the reviewers. Any product that may be evaluated in this article, or claim that may be made by its manufacturer, is not guaranteed or endorsed by the publisher.
