# Supplementary material for: Evaluation of Th2 and Th17 Immunity-Related Factors as Indicators of Brucellosis
Source: Front Cell Infect Microbiol. 2022 Jan 7;11:786994. doi: 10.3389/fcimb.2021.786994 (PMC8777051; doi:10.3389/fcimb.2021.786994)
Supplement: Supplementary file 1 [file DataSheet_1.pdf]

Supplementary Data

Evaluation of Th2 and Th17 immunity-related factors as indicators of brucellosis

Reza Gheitasi[1,4], Fariba Keramat[3], Sara Khosravi[2], Mehrdad Hajilooi[4], Mathias W. Pletz[1], Oliwia Makarewicz[1]

[1] Institute for Infectious Diseases and Infection Control, Jena University Hospital, Jena, Germany.
[2] Department of Microbiology, School of Medicine, Hamadan University of Medical Sciences, Hamadan, Iran.
[3] Brucellosis Research Center, Hamadan University of Medical Sciences, Hamadan, Iran.
[4] Department of Immunology, School of Medicine, Hamadan University of Medical Sciences, Hamadan, Iran.

Running title: TRAF3IP2: An indicator in brucellosis

Table S1. Primers and targets used in this study

Gene Name	Forward primer (5′ to 3′)	Reverse primer (5′ to 3′)
IL-25	GGAGATATGAGTTGGACAGAGAC	AGCTAAGGAAACACGGTACAG
IL-17RB	CTTATCCAACACAGCACTATCATC	ACCGTAGCACCTTCACTATCC
TRAF3IP2	ACCGTGATGATAATCGTAGCAATC	CTGTAGACATGAGTGTTCTGAAGC
Th2-LCR lncRNA	TGCAGCGTTACTTGGGTGAGTTG	CCGAGTGGTGATGCTGAAGGGA
18S-rRNA	GTAACCCGTTGAACCCCATT	CCATCCAATCGGTAGTAGCG

Table S2. Means and standard deviations and normality statistic of the ct-values of the 18S-rRNA and the Δct-values of the target genes qPCR.

		Acute	Relapse	Treated	Control
18S-rRNA	Mean	11.02	10.35	10.04	10.47
	Std. Deviation	1.515	1.858	1.493	1.800
	Shapiro-Wilk test statistic (W)	0.9590	0.9431	0.9441	0.9491
	Shapiro-Wilk test P-value	0.2424	0.0835	0.0897	0.1602
IL-25	Mean	18.00	18.45	19.23	20.11
	Std. Deviation	2.223	2.218	2.078	2.970
	Shapiro-Wilk test statistic (W)	0.9534	0.9232	0.9622	0.9597
	Shapiro-Wilk test P-value	0.1803	0.0253	0.3710	0.3634
IL-17RB	Mean	16.93	17.74	18.87	18.77
	Std. Deviation	2.990	3.029	2.288	2.484
	Shapiro-Wilk test statistic (W)	0.9303	0.9722	0.9527	0.9425
	Shapiro-Wilk test P-value	0.0358	0.5620	0.1717	0.1167
TRAF3IP2	Mean	12.79	15.23	15.75	16.37
	Std. Deviation	3.938	3.197	3.054	2.802
	Shapiro-Wilk test statistic (W)	0.8476	0.9413	0.9686	0.9894
	Shapiro-Wilk test P-value	0.0003	0.0739	0.4432	0.9877
Th2-LCR lncRNA	Mean	15.63	15.70	17.93	18.80
	Std. Deviation	3.371	3.479	3.076	3.106
	Shapiro-Wilk test statistic (W)	0.9481	0.8971	0.9632	0.9883
	Shapiro-Wilk test P-value	0.1386	0.0045	0.3172	0.9819

Table S3 Mean $2^{-\triangle\triangle Ct}$ values and the standard deviation (SD) of all analyzed genes in all groups

	IL-25		IL-17RB		TRAF3IP2		Th2-LCR lncRNA	
	Mean	SD	Mean	SD	Mean	SD	Mean	SD
Acute	9.79	10.79	19.43	31.44	63.03	71.93	56.35	104.42
Relapse	6.79	7.10	10.46	19.12	23.70	60.53	47.83	74.56
Treated	3.93	4.51	2.33	3.28	11.63	32.76	10.64	18.07
Control	5.95	11.63	4.18	12.10	5.34	14.23	8.76	22.20

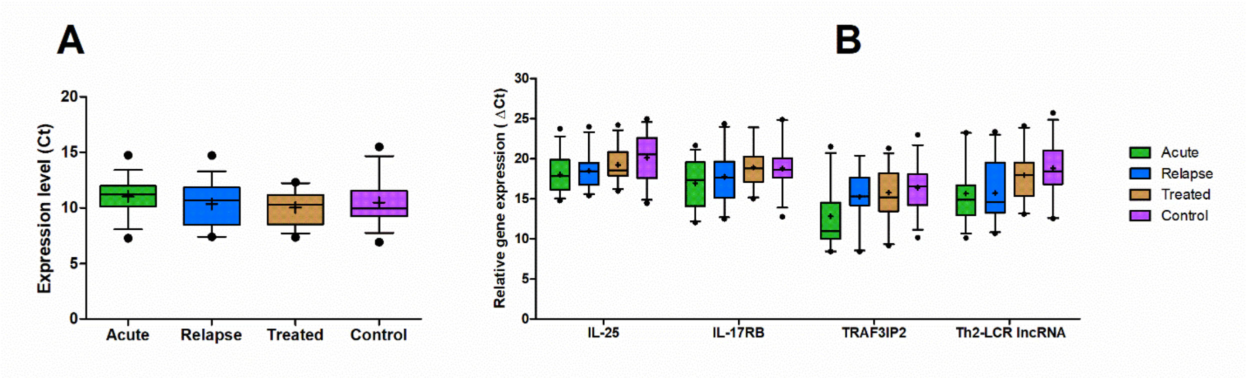

Figure S1 Ct Values of the 18S-rRNA (A) and ΔCt-values of the four target genes (B) in the different study groups (differentiated by the color). The boxes indicate the quartiles (25 % to 75 %) and the whiskers the range of the values from 5 % to 95 %. The line an (+) in the box indicate the median and mean, respectively. The dots indicate the outliers.
